# Rapid maxillary expansion treatment increases mid-facial depth in early mixed dentition

**DOI:** 10.3389/fped.2022.1028968

**Published:** 2023-02-01

**Authors:** Jiaping Si, Xinyi Hu, Yu Du, Mengyao Wei, Lehan Xu, Bing Li, Xiaoyan Chen, Xuan Li

**Affiliations:** ^1^ Stomatology Hospital, School of Medicine, Zhejiang University, Hangzhou, China; ^2^ Department of Stomatology, Children's Hospital of Fudan University, National Children's Medical Center, Shanghai, China; ^3^ Department of Orthodontics, Shanghai Stomatological Hospital & School of Stomatology, Fudan University, Shanghai, China; ^4^ Shanghai Key Laboratory of Craniomaxillofacial Development and Diseases, Fudan University, Shanghai, China

**Keywords:** rapid maxillary expansion, early mixed dentition, facial depth, maxillary sinus, nasal cavity, cone-beam computer tomography

## Abstract

**Objective:**

To evaluate the effects of rapid maxillary expansion (RME) on mid-facial depth in early mixed dentition and to investigate the relationship between change in mid-facial depth and maxillary sinus and nasal cavity.

**Methods:**

A total of 35 patients with mixed dentition treated with a Haas expander were included in this retrospective study. All patients underwent a cone-beam computed tomography scan before and after rapid maxillary expansion. The Wilcoxon signed-rank test was performed to evaluate the changes in maxillary width, facial depth, maxillary sinus, and nasal cavity volume before and after expansion. Multiple linear regression analysis was applied to evaluate the correlations among them.

**Results:**

The hard and soft tissue facial depth in the middle third increased significantly (*P* < 0.001). The gain on the outer sagittal plane (1.04–1.52 mm) was slightly bigger than that on the inner sagittal plane (0.91–1.30 mm). Maxillary width and nasal cavity width increased 3.42 ± 0.93 mm (*P* < 0.001) and 2.25 ± 0.77 mm (*P* < 0.001), respectively, after treatment. A gain was also achieved in both nasal cavity volume (2,236.15 mm^3^, *P* < 0.001) and maxillary sinus volume (1,227.33 mm^3^, *P* < 0.001). Multiple linear regression analysis showed that with the increase in maxillary sinus volume, the facial depth increased as well (*B* = 0.455–0.683, *P* < 0.05). Also, statistically significant correlations were found between nasal width and nasal cavity volume (*B* = 0.384, *P* < 0.05).

**Conclusion:**

The depth of the middle third face increased significantly. The facial depth increase was related to the enlargement of maxillary sinus volume, while the nasal cavity volume gain was related to the nasal width increase. This indicated that RME might enhance the fullness of the mid-face and facilitate the patency of nose breathing.

## Introduction

A constricted maxilla is commonly found in all age groups, having an especially high prevalence of 18% in children with mixed dentition (1). It often manifests as decreased posterior overjet and unilateral or bilateral posterior crossbite, which may further lead to anteroposterior discrepancy and facial asymmetry in later developmental stages (2).

Rapid maxillary expansion (RME) has been widely used to correct constricted maxillas. Recent studies have suggested that RME could improve nasal morphology and increase nasal cavity and maxillary sinus volume (MSV) (3–6). In addition, it has been reported that maxillary transverse expansion might positively affect respiratory function and improve oxygen saturation (7).

It is equally important to understand the effect of RME on facial hard and soft tissue. In recent years, several clinical data have revealed that nasal width, alar base width, and mouth width increase significantly after RME treatment (8, 9). Lagravere et al. argued that the maxilla moves forward after RME, although these findings were not clinically important (10). However, most previous studies focused on the midline facial region of adults, while only a few studies examined the effects of bilateral facial features on growing patients.

With the development of imaging, it was suggested that the growth of the maxillary sinus was proportional to the growth of maxillofacial bones (11). However, thus far, no study has evaluated the relationship between sagittal facial dimensions and maxillary sinus volume improvement produced by RME treatment. Therefore, this study aims to evaluate the effects of RME on mid-face in early mixed dentition by cone-beam computed tomography (CBCT) and to explore the relationship between mid-facial change and maxillary sinus and nasal cavity.

## Materials and methods

### Subjects

This retrospective study was approved by the Children’s Hospital of Fudan University committee (approval No. 2021253), and informed consent was obtained from subjects and their parents. A total of 35 children, 19 boys and 16 girls, with a mean age of 8.2 years (ranging from 6.5 to 10 years), who underwent RME with a Haas expander between June 2019 and June 2021, were included.

The inclusive criteria were the following: (1) patients with a maxillary transverse deficiency of more than 5 mm; (2) patients aged 6–10 years with erupted first permanent molars; (3) those with more than two-third of roots of primary canines and primary second molars.

The exclusion criteria were the following: (1) patients with systemic diseases, craniofacial defects, or trauma; (2) patients with rhinitis or respiratory diseases; (3) patients with a history of orthodontic treatments.

### CBCT scan protocol

CBCT was obtained for initial diagnosis (T0) and 6 months’ retention after expansion (T1). All CBCT scans were acquired from a KaVo 3DeXam CBCT machine (KaVo OP 3D Vision, Biberach, Germany) by one operator. The parameter settings of CBCT were field of view (17 × 23 cm^2^), tube voltage tube current (95 kV), tube current (5 mA), voxel size (200 μm), and scan time (15 s). The patients were instructed to remain at an upright position with a centric occlusion and the Frankfort horizontal (FH) plane paralleling to the ground.

### Haas expander appliance

**Figure 1:**
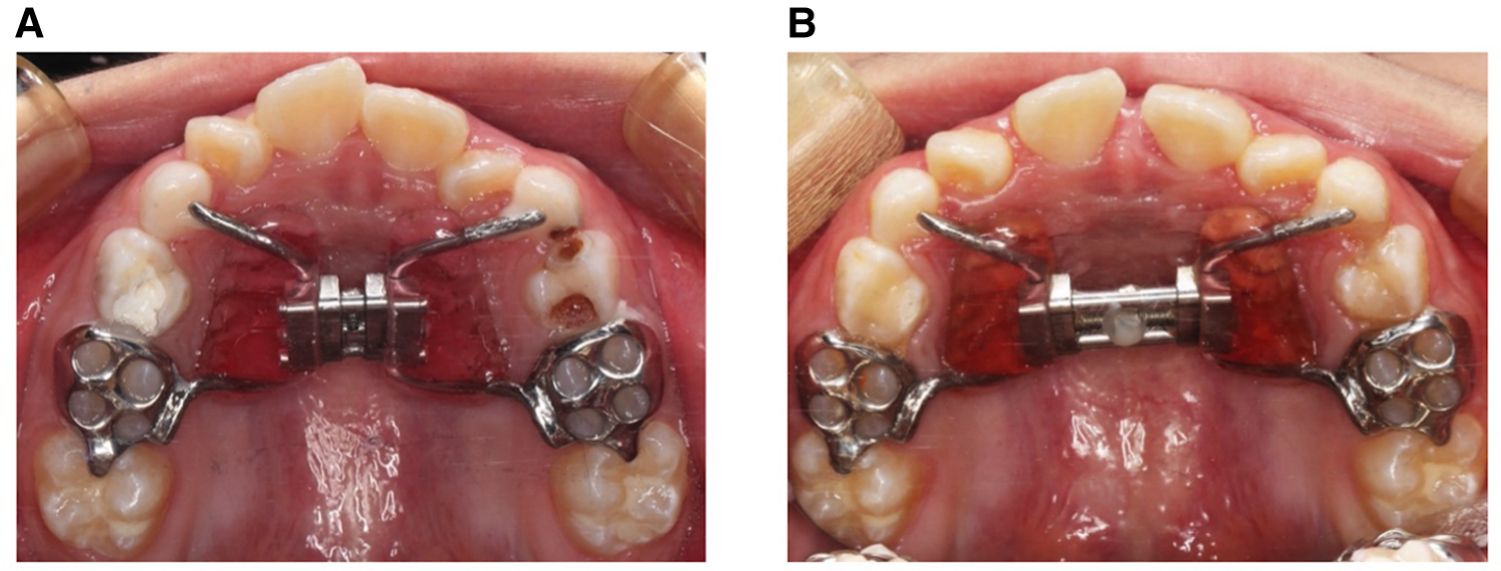
Haas expander appliance and facial profile. (**A**) Occlusal view of the Haas expander before RME treatment. (**B**) Occlusal view of the expander after RME treatment.

All patients were treated with a Haas expander appliance made by the same technician group with the same design (Figure 1). Maxillary expansion was initiated when the Haas expander was in place. The expander was activated twice daily (0.20 mm/turn) for the first 7 days and once daily for another 14 days. Also, patients were instructed to activate the expansion at the same time every day. After expansion, the patients kept the appliance for 6 months as retention.

### Measurements

**Figure 2:**
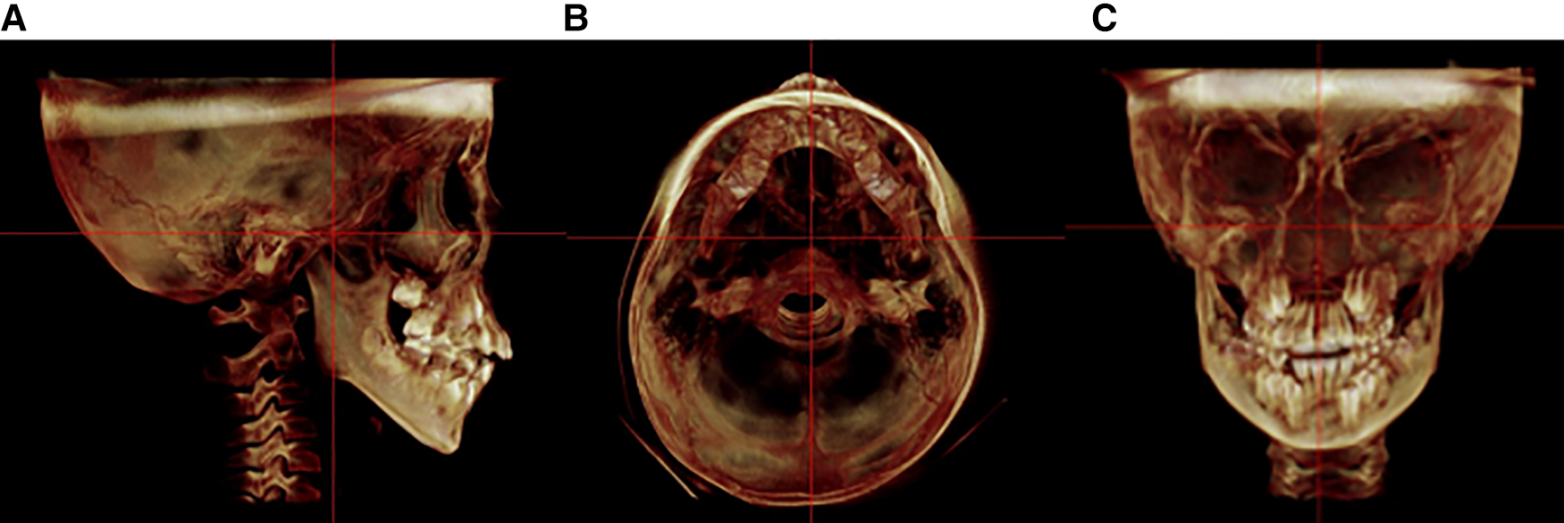
Standardized orientation of CBCT for measurement. (A) Sagittal plane. (B) Horizontal plane. (C) Coronal plane.

All CBCT scans were saved as Dicom files and then exported to the Dolphin Imaging software (version 11.9, Dolphin Imaging and Management Solutions, Chatsworth, CA, United States). The three-dimensional models were then adjusted by following three reference planes: (1) horizontal plane: Frankfort plane; (2) sagittal plane: a plane perpendicular to the FH plane and running through the crista galli to the basion; (3) coronal plane: a plane perpendicular to the FH plane and running through the center of the sella (Figure 2).

**Figure 3:**
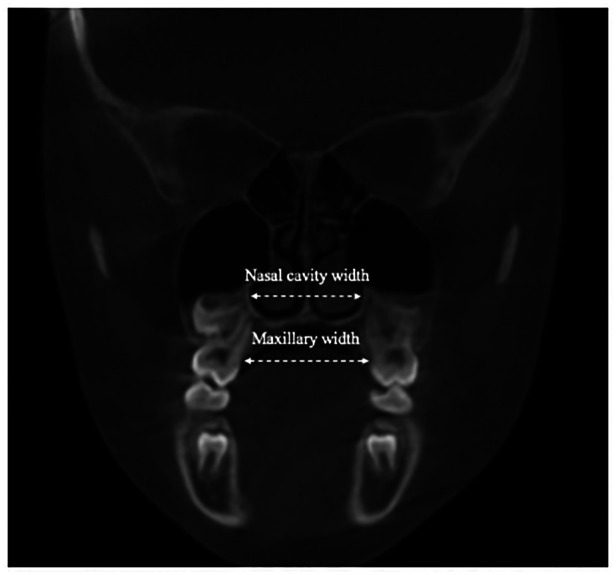
The coronal section on the first molars. The maxillary and nasal width are measured from the palatal alveolar ridge of the first molar to the other side.

The maxillary width was measured through the bilateral palatal alveolar crest in the region of the first molars. The nasal width was determined from the bilateral widest transverse portion of the nasal cavity in the region of the first molars (Figure 3).

**Figure 4:**
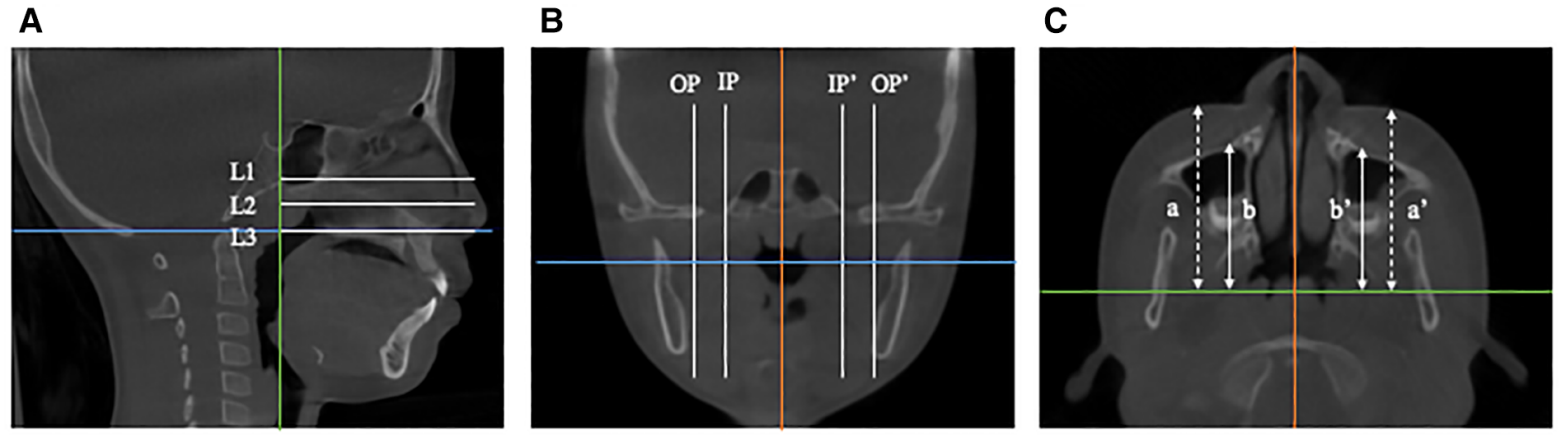
Measurements of the mid-facial depth. (A) Sagittal plane.The horizontal planes are paralleled to the FH plane, including the FH plane (L1), the plane through the anterior nasal spine (L3), and the plane (L2) bisecting L1 and L3. (B) Coronal plane. The bilateral inner (IP) and outer (OP) sagittal planes extend through the orbitale and the outermost rim of the sella, respectively. (C) Horizontal plane. The dotted line (a and a') represents soft tissue facial depth, and the solid line (b and b') denotes hard tissue facial depth.

The measurements of mid-facial depth were referenced to Kim et al. (12). Four sagittal planes and three horizontal planes were identified as follows to evaluate the mid-facial depth. The bilateral inner (IP) and outer (OP) sagittal plane extended through the orbitale and the outermost rim of the sella, respectively. The horizontal planes were paralleled to the FH plane, including the FH plane (L1), the plane through the anterior nasal spine and paralleling to the FH plane (L3), and the plane (L2) bisecting L1 and L3. The facial depth of hard tissue and soft tissue was measured from the anterior edge of the maxilla and soft tissue to the coronal reference plane on three horizontal planes (Figure 4).

**Figure 5:**
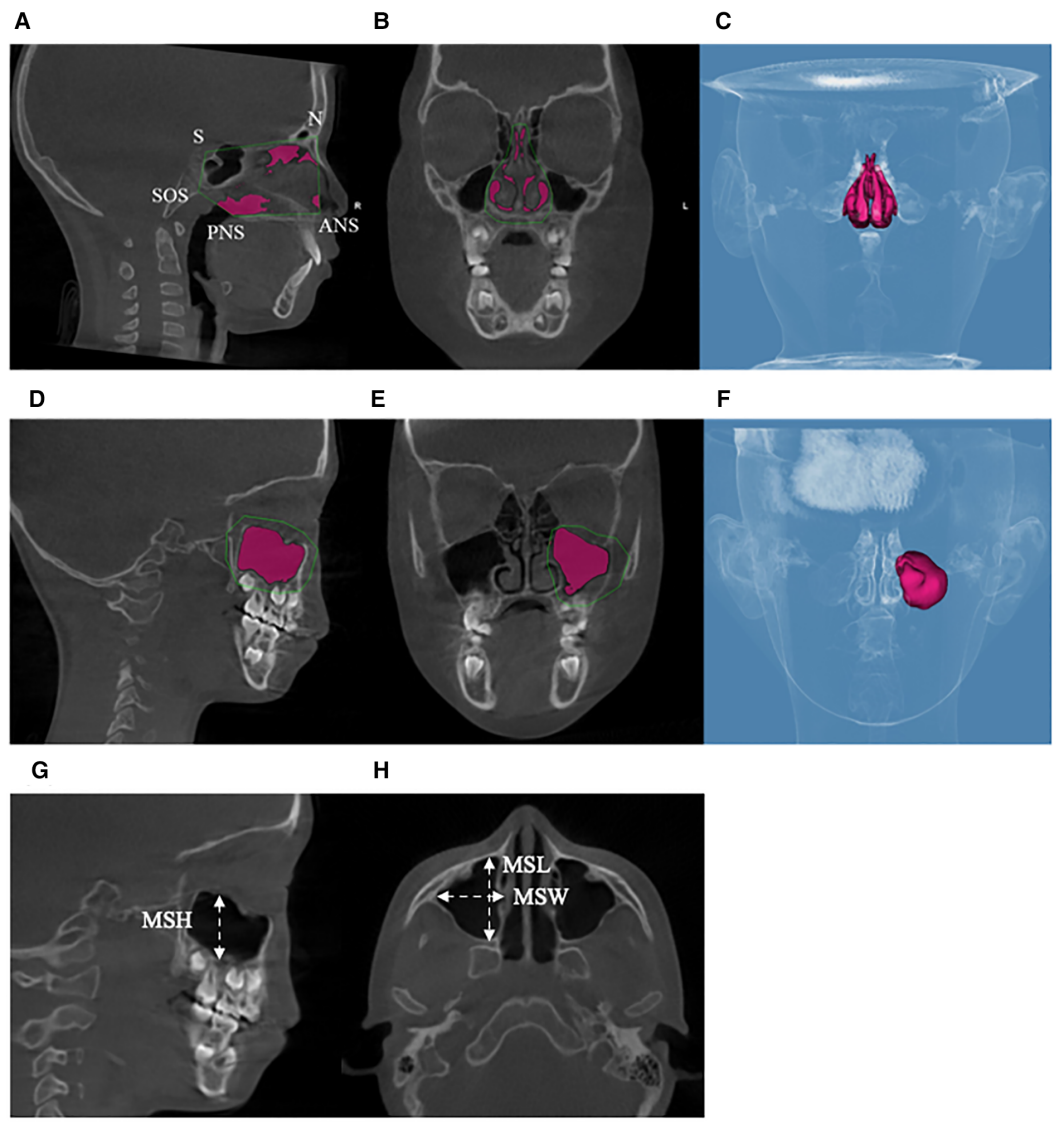
Measurements of the maxillary sinus and nasal cavity. (**A–C**) Maxillary sinus boundaries and maxillary sinus volume. (**D–F**) Nasal cavity boundaries and nasal cavity volume. (**G**) The height of the maxillary sinus. (**H**) The width of the maxillary sinus and the length of the maxillary sinus.

The measurements of the maxillary sinus were referenced to the previously reported methods by Hamdy and Abdel-Wahed (13). Dolphin Software 3D rendering was used to measure the maxillary sinus. First, the borders of the maxillary sinus were defined, and the threshold value was set as 55 to gain the largest measurement. Subsequently, the MSV was automatically calculated through semiautomatic segmentation. The height of the maxillary sinus (MSH) was measured from the lowest point of the cortical boundary of the orbital floor to the most inferior border of the cortical boundary of the sinus floor. The width (MSW) and the length (MSL) of the maxillary sinus were measured as the maximum mediolateral and anteroposterior dimensions on the cross section along the root of the zygoma on each side, respectively (Figure 5). In order to measure the nasal cavities, the following were demarcated: nasion, anterior nasal spine, posterior nasal spine, sella, and the most inferior point of the spheno-occipital synchondrosis (Figure 5).

### Statistical analysis

All images were measured by two trained examiners, and intraclass correlation efficiency was used to test intra-observer reliability. The Shapiro–Wilk test was carried out to evaluate the distribution of the dependent variable, and the results showed abnormal data distribution. Therefore, the Wilcoxon signed-rank test was performed to evaluate the changes in maxillary width, facial depth, maxillary sinus, and the nasal cavity between T0 and T1. In addition, multiple linear regression analysis was applied to evaluate the correlations between facial depth change and the following four variables: ΔMSV, ΔNV, ΔNW, and ΔMW. This analysis was also conducted to determine whether ΔNV was related to ΔMSV, ΔNW, and ΔMW. All data were collected with SPSS statistical software (version 23.0, IBM Corp, Armonk, NY, United States); *P* < 0.05 indicated statistical significance.

## Results

Because there was no difference between the left and the right sides, the average value of both sides was used for analysis. In all sagittal and horizontal planes, the facial depth increment of hard tissue (1.10–1.52 mm) was slightly greater than that of soft tissue (0.91–1.36 mm). In the horizontal plane, the increment on the L1 plane (0.91–1.13 mm) was lower than that on the L2 plane (1.05–1.38 mm), whereas the greatest increase was observed on the L3 plane (1.22–1.52 mm). In the sagittal plane, all measurements of mid-facial depth showed a significant increase after RME treatment (*P* < 0.001) (Table 1). The average mid-facial depth increment on the outer sagittal plane (1.28 mm) was approximately 0.35 mm higher than that on the inner sagittal plane (0.93 mm).

**Table 1:** Comparison of the mid-facial depth changes between T0 and T1.

Measurements		T0		T1		*P*-value	ΔT1–T0	
		Mean	SD	Mean	SD		Mean	SD
**Outer facial depth (mm)**								
L1	Hard tissue	53.02	2.78	54.15	2.85	0.000*	1.13	1.17
	Soft tissue	59.63	3.25	60.67	3.16	0.000*	1.04	1.27
L2	Hard tissue	50.89	3.07	52.27	3.10	0.000*	1.38	1.40
	Soft tissue	61.68	3.25	62.95	2.86	0.000*	1.27	1.24
L3	Hard tissue	50.19	3.79	51.71	3.91	0.000*	1.52	1.70
	Soft tissue	62.46	3.41	63.82	2.83	0.000*	1.36	1.41
**Inner facial depth (mm)**								
L1	Hard tissue	50.97	2.88	52.10	2.82	0.000*	1.13	0.99
	Soft tissue	58.35	3.62	59.26	3.44	0.000*	0.91	1.17
L2	Hard tissue	48.00	2.82	49.10	2.77	0.000*	1.10	1.03
	Soft tissue	60.68	3.70	61.73	3.46	0.000*	1.05	1.41
L3	Hard tissue	43.80	3.45	45.10	3.79	0.000*	1.30	1.66
	Soft tissue	62.11	3.75	63.33	3.38	0.000*	1.22	1.20

L1, FH plane; L2, the plane bisecting L1 and L3; L3: the plane through the anterior nasal spine and paralleling to the FH plane; T0, before treatment; T1, 6 months after retention.

* Wilcoxon signed-rank test: P ≤ 0.05.

For volumetric analysis, maxillary width and nasal cavity width were increased by 3.42 ± 0.93 mm and 2.25 ± 0.77 mm after RME (*P* < 0.001). A significant enlargement was found in the nasal cavity volume (2,236.15 mm^3^, *P* < 0.001). The length, width, and height of the maxillary sinus significantly increased by 0.78, 1.37, and 1.01 mm, respectively. Thus, the maxillary sinus volume showed a significant increase after RME treatment (1,227.33 mm^3^, *P* < 0.001) (Table 2).

**Table 2:** Comparison of the maxillary width, nasal cavity, and maxillary sinus changes between T0 and T1.

Measurements	T0		T1		*P*-value	ΔT1–T0	
	Mean	SD	Mean	SD		Mean	SD
Maxillary width (mm)	31.23	2.30	34.41	2.58	0.000*	3.42	0.93
Nasal cavity width (mm)	25.45	1.77	27.86	1.85	0.000*	2.36	0.62
Nasal cavity (mm^3^)	12471.48	2924.45	14708.03	3205.38	0.000*	2236.55	280.93
Maxillary sinus volume (mm^3^)	10394.22	2509.42	11621.55	2608.10	0.000*	1227.33	781.29
Maxillary sinus length (mm)	33.93	2.36	34.71	2.44	0.000*	0.78	1.11
Maxillary sinus width (mm)	25.85	3.50	27.22	3.42	0.000*	1.37	1.15
Maxillary sinus height (mm)	25.06	2.62	26.07	2.75	0.000*	1.01	1.31

T0, before treatment; T1, 6 months after retention.

* Wilcoxon signed-rank test: P ≤ 0.05.

According to the multiple regression analysis, for every 1,000 mm^3^ of the maxillary sinus volume increase, the hard tissue facial depth enlarged by 0.477–0.683 mm, and the soft tissue facial depth enlarged by 0.455–0.629 mm. With the increase in the maxillary width of the inner plane, the hard tissue facial depth also enlarged, and the correlation coefficients varied from 0.352 to 0.439 (Table 3). In addition, nasal cavity volume was significantly associated with nasal width (*B* = 0.384, *P* < 0.05), whereas other variables had no significant effect on nasal cavity volume (Table 4).

**Table 3:** Multiple linear regression analysis for facial depth change.

Measurements		ΔMSV (mm^3^)		ΔNV (mm^3^)		ΔMW (mm)		ΔNW (mm)	
		Beta	*P*-value	Beta	*P*-value	Beta	*P*-value	Beta	*P*-value
**Δ Outer facial depth (mm)**									
L1	Hard tissue	0.598	0.003*	−0.114	0.548	0.261	0.13	−0.171	0.325
	Soft tissue	0.455	0.030*	−0.177	0.394	0.237	0.202	−0.146	0.438
L2	Hard tissue	0.683	0.001*	−0.243	0.186	0.23	0.161	−0.15	0.364
	Soft tissue	0.617	0.002*	−0.187	0.333	0.176	0.308	−0.125	0.473
L3	Hard tissue	0.592	0.004*	−0.228	0.244	0.188	0.277	−0.023	0.895
	Soft tissue	0.615	0.002*	−0.194	0.316	0.113	0.509	−0.019	0.915
**Δ Inner facial depth (mm)**									
L1	Hard tissue	0.477	0.013*	−0.184	0.33	0.439	0.013*	−0.217	0.209
	Soft tissue	0.623	0.002*	−0.222	0.237	0.29	0.087	−0.094	0.578
L2	Hard tissue	0.530	0.007*	−0.096	0.616	0.352	0.045*	−0.29	0.102
	Soft tissue	0.591	0.003*	−0.271	0.156	0.302	0.079	−0.144	0.401
L3	Hard tissue	0.637	0.001*	−0.013	0.942	0.362	0.026*	−0.261	0.108
	Soft tissue	0.629	0.001*	−0.326	0.085	0.259	0.124	−0.076	0.649

L1, FH plane; L2, the plane bisecting L1 and L2; L3: the plane through the anterior nasal spine and parallel to the FH plane; MSV, maxillary sinus volume; NV, nasal volume; MW, maxillary width; NW, nasal width; Beta, standardized coefficient.

^*^ *P* ≤ 0.05.

**Table 4:** Multiple linear regression analysis for nasal cavity change.

Measurements	ΔMSV (mm^3^)		ΔMW (mm)		ΔNW (mm)	
	Beta	*P*	Beta	*P*	*B*	*P*
ΔNV (mm^3^)	0.313	0.071	−0.296	0.088	0.384	0.039*

MSV, maxillary sinus volume; NV, nasal volume; MW, maxillary width; NW, nasal width; Beta, standardized coefficient.

^*^P ≤ 0.05.

## Discussion

Transversal maxillary deficiency may cause masticatory disorders and jaw dysfunction, which, in turn, could affect the health of oral and jaw development and facial esthetics (14). RME is an effective treatment used to expand the mid-palatal suture in growing patients; however, the right age for expansion remains debatable (15). Some scholars have claimed that RME should be performed at an early age because the potential for growth will be more and it could induce more transversal skeletal changes (16). Yet, when the expander is used in preschool children, it exerts substantial expansion pressure that might induce a flattened nasal shape and nosebleed (17). Therefore, the age of patients in the present study ranged from 6 to 10 years to avoid the negative effects of RME. In our investigation, although the tooth-borne RME was used, a significant expansion of nasal and maxilla was obtained, thus confirming that RME helps achieve a noticeable improvement in transversal skeletal changes. However, the significant skeletal effects obtained could be strongly related to the age of the subjects included in our study, and it is possible that they would be less significant in the late pubertal stage or in adolescence (18, 19). In addition, previous studies showed that RME in early mixed dentition might spontaneously improve molar relationships and the sagittal skeletal pattern, leading to a better growth pattern (20).

The abnormal facial features of transversal maxillary deficiency include a deficiency of the mid-face and deepened nasolabial folds (21). Also, the effects of maxillary expansion were limited not only to the maxilla but also to the surrounding facial region. As reported by Altorkat et al., RME produced a slight increase in nasal tip protrusion, but there was no statistical significance (22). Baysal et al. (8) found that alar base enlargement was one of the consequences of RME due to the weak correlations between hard and soft tissue. Altındiş et al. (23) revealed that modified acrylic splint RME appliances made the upper lip more protrusive. Although several studies have assessed the effects of RME on facial hard and soft tissue, most of them focused on the midline area. The present study investigated the mid-face change after RME, finding that all measurements of mid-facial depth improved remarkably. The enlargement of facial depth was similar in different sagittal planes, while it varied slightly in different horizontal planes. The closer to the palatal plane, the more increase in facial depth, which might indicate the effectiveness of RME on the maxillofacial region, which is consistent with the pyramid model.

Normal growth should be considered when facial depth increases after RME. Previous studies indicated that the mean increase in mid-facial depth in children aged 7–9 years was approximately 0.5 mm per half-year (24). Longo reported that the increase in the normal growth of facial depth in 6 months was irrelevant (25). In our study, we found that hard and soft tissue facial depth increased by 1.1–1.52 and 0.91–1.38 mm in 6 months, respectively. Consequently, there may be an additional increase resulting from RME. This additional growth may be directly affected by RME and will be probably followed by airway improvement, promoting a temporary growth development of the mid-face. Our study also evaluated the upper airway changes, finding a significant increase in the airway depth and volume. These results indicated that RME had beneficial effects on the upper respiratory tract, which are consistent with those of previous studies (6, 26).

To further elucidate the causes of mid-facial changes, we measured the maxillary sinus and nasal cavity, finding a significant increase in maxillary sinus volume and nasal cavity volume. Also, the length, height, and width of the maxillary sinus were measured, and significant increases were observed. Lorkiewicz-Muszyńska et al. divided 170 patients into 17 groups based on their age to evaluate the age-related changes in the dimensions of the maxillary sinus (27), finding that the length, width, height, and volume of the maxillary sinus in children aged 6–10 years increased by approximately 0.47 mm, 0.53 mm, 0.55 mm, and 537 mm^3^ per 6 months, respectively. However, in our study, the above measurements increased at least by 0.78 mm, 1.37 mm, 1.01 mm, and 1277.33 mm^3^, respectively. This indicated that RME could induce a higher growth of the maxillary sinus, especially in maxillary sinus width, which is consistent with the findings of the previous studies (2).

Multiple linear regression analysis showed that maxillary sinus volume, followed by maxillary width, contributed the most to mid-facial depth, while other variables seemed to have no significant effect on facial depth. A reasonable estimation of this result was that enlarged maxillary width contributed to the increase in nasal cavity volume, further reducing respiratory resistance and promoting generalized and maxillary sinus growth (6, 28). In addition, maxillary width was the other variable that could enlarge the hard tissue’s facial depth at the inner plane. A hypothesis was that the palatal expansion induced the compression of the maxillary sinus, thus resulting in the skeletal reorganization of the maxillary sinus and enlargement of the maxillary sinus length. According to the regression analysis, with the widening nasal width, there was a higher increase in nasal cavity volume (*B* = 0.384, *P* < 0.05). Still, the change in the maxillary width was not significantly correlated with the nasal cavity volume change, which could be due to the teeth's inclination toward the buccal aspect resulting in a different expansion of the nasal cavity and maxillary alveolar bone.

In summary, management considerations for maxillofacial problems in growing adults are keys for interceptive treatment. This study indicated that RME could improve the constricted maxilla, increase the maxillary sinus volume and nasal cavity volume, reduce respiratory resistance, and promote the growth and development of mid-facial depth. We suggested that RME had a positive role in mid-facial growth management and could lead to better facial esthetics in early mixed-dentition patients (29).

However, this study still has some limitations. It did not contain a control group because of ethical reasons, and therefore, how much of the detected differences could be attributed to natural growth (not RME) was unknown (30). In addition, the follow-up period appeared to be comparatively short than other studies and the sample size was relatively small. Thus, we plan to increase the sample size in our future study and carry out a stratified analysis of people in different age groups to further clarify the effect of RME on the fullness of the middle third of the face. Also, the patients included in this study will be followed up for a longer time to further track their mid-face changes. Furthermore, it is hoped that more reliable three-dimensional digital diagnostic software could improve the accuracy of measurement (31), and artificial intelligence (AI) and deep learning paradigm could be used to achieve full automatic segmentation of maxillary sinus volume (32).

## Conclusion

The depth of the middle third of the face increased significantly and the volume of the maxillary sinus and nasal cavity enlarged in early mixed-dentition patients treated by RME. The facial depth increase was related to the enlargement of the maxillary sinus volume, while the nasal cavity volume gain was related to the nasal width increase. This indicated that RME might enhance the fullness of the mid-face and facilitate the patency of nose breathing.

## Data Availability

The raw data supporting the conclusions of this article will be made available by the authors, without undue reservation.

## References

[1] Carlson C Sung J McComb RW MacHado AW Moon W. Microimplant-assisted rapid palatal expansion appliance to orthopedically correct transverse maxillary deficiency in an adult. Am J Orthod Dentofac Orthop. (2016) 149(5):716–28. 10.1016/j.ajodo.2015.04.043 27131254

[2] Erdur EA Yıldırım M Karatas RMC Akin M. Effects of symmetric and asymmetric rapid maxillary expansion treatments on pharyngeal airway and sinus volume. Angle Orthod. (2020) 90(3):425–31. 10.2319/050819-320.1 33378426 PMC8032301

[3] Kavand G Lagravère M Kula K Stewart K Ghoneima A. Retrospective CBCT analysis of airway volume changes after bone-borne vs tooth-borne rapid maxillary expansion. Angle Orthod. (2019) 89(4):566–74. 10.2319/070818-507.1 30768911 PMC8117203

[4] Truong CT Jeon HH Sripinun P Tierney A Boucher NS. Short-term and long-term effects of rapid maxillary expansion on the nasal soft and hard tissue. Angle Orthod. (2021) 91(1):46–53. 10.2319/022320-120.1 33289784 PMC8032292

[5] Silva Filho OG Lara TS Ayub PV Ohashi AS Bertoz FA. Photographic assessment of nasal morphology following rapid maxillary expansion in children. J Appl Oral Sci. (2011) 19(5):535–43. 10.1590/s1678-77572011000500017 21986660 PMC3984203

[6] Fastuca R Meneghel M Zecca PA Mangano F Antonello M Nucera R Multimodal airway evaluation in growing patients after rapid maxillary expansion. Eur J Paediatr Dent. (2015) 16(2):129–34. PMID: 26147819 26147819

[7] Fastuca R Perinetti G Zecca PA Nucera R Caprioglio A. Airway compartments volume and oxygen saturation changes after rapid maxillary expansion: a longitudinal correlation study. Angle Orthod. (2015) 85(6):955–61. 10.2319/072014-504.1 26516709 PMC8612044

[8] Baysal A Ozturk MA Sahan AO Uysal T. Facial soft-tissue changes after rapid maxillary expansion analyzed with 3-dimensional stereophotogrammetry: a randomized, controlled clinical trial. Angle Orthod. (2016) 86:934–42. 10.2319/111315-766.1 27058647 PMC8597340

[9] Huang J Li CY Jiang JH. Facial soft tissue changes after nonsurgical rapid maxillary expansion: a systematic review and meta-analysis. Head Face Med. (2018) 14(1):6. 10.1186/s13005-018-0162-8 29562914 PMC5863368

[10] Lagravere MO Major PW Flores-Mir C. Long-term skeletal changes with rapid maxillary expansion: a systematic review. Angle Orthod. (2005) 75:1046–52. 10.1043/0003-3219(2005)75 16448254

[11] Whyte A Boeddinghaus R. The maxillary sinus: physiology, development and imaging anatomy. Dentomaxillofac Radiol. (2019) 48(8):20190205. 10.1259/dmfr.20190205 31386556 PMC6951102

[12] Kim B Lee HC Kim SH Kim Y Son W Kim SS. Correction to: hard- and soft-tissue profiles of the midface region in patients with skeletal class III malocclusion using cone-beam computed tomography multiplanar-reconstructed image analysis. Korean J Orthod. (2018) 48(5):346. 10.4041/kjod.2018.48.5.346 30206534 PMC6123072

[13] Hamdy RM Abdel-Wahed N. Three-dimensional linear and volumetric analysis of maxillary sinus pneumatization. J Adv Res. (2014) 5(3):387–95. 10.1016/j.jare.2013.06.006 25685506 PMC4294758

[14] Andrucioli M Matsumoto M. Transverse maxillary deficiency: treatment alternatives in face of early skeletal maturation. Dental Press J Orthod. (2020) 25(1):70–9. 10.1590/2177-6709.25.1.070-079 32215481 PMC7077945

[15] Baccetti T Franchi L Cameron CG McNamara JA Jr. Treatment timing for rapid maxillary expansion. Angle Orthod. (2001) 71(5):343–50. 10.1043/0003-3219 11605867

[16] Dugoni SA. Comprehensive mixed dentition treatment. Am J Orthod Dentofacial Orthop. (1998) 113(1):75–84. 10.1016/S0889-5406(98)70278-1 9457021

[17] Proffit WR White RP Sarver DM. Contemporary treatment of dentofacial deformity. St. Louis, MO: Mosby (2003).

[18] Leonardi R Ronsivalle V Lagravere MO Barbato E Isola G Giudice AL. Three-dimensional assessment of the spheno-occipital synchondrosis and clivus after tooth-borne and bone-borne rapid maxillary expansion. Angle Orthod. (2021) 91(6):822–9. 10.2319/013021-86.1 34129666 PMC8549551

[19] Lo Giudice A Ronsivalle V Lagravere M Leonardi R Martina S Isola G. Transverse dentoalveolar response of mandibular arch after rapid maxillary expansion (RME) with tooth-borne and bone-borne appliances: a CBCT retrospective study. Angle Orthod. (2020) 1(90):680–7. 10.2319/042520-353.1 PMC8032272 33378488

[20] Garrett BJ Caruso JM Rungcharassaeng K Farrage JR Kim JS Taylor GD. Skeletal effects to the maxilla after rapid maxillary expansion assessed with cone-beam computed tomography. Am J Orthod Dentofacial Orthop. (2008) 134(1):8–9. 10.1016/j.ajodo.2008.06.004 18617096

[21] Zupan J Ihan Hren N Verdenik M. An evaluation of three-dimensional facial changes after surgically assisted rapid maxillary expansion (SARME): an observational study. BMC Oral Health. (2022) 22(1):155. 10.1186/s12903-022-02179-1 35501780 PMC9063160

[22] Altorkat Y Khambay BS McDonald JP Cross DL Brocklebank LM Ju X. Immediate effects of rapid maxillary expansion on the naso-maxillary facial soft tissue using 3D stereophotogrammetry. Surgeon. (2016) 14:63–8. 10.1016/j.surge.2014.04.005 24947501

[23] Altındiş S Toy E Başçiftçi FA. Effects of different rapid maxillary expansion appliances on facial soft tissues using three-dimensional imaging. Angle Orthod. (2016) 86:590–8. 10.2319/051115-319.1 26381118 PMC8601490

[24] Blanchette ME Nanda RS Currier GF Ghosh J Nanda SK. A longitudinal cephalometric study of the soft tissue profile of short- and long-face syndromes from 7 to 17 years. Am J Orthod Dentofacial Orthop. (1996) 109(2):116–31. 10.1016/s0889-5406(96)70172-5 8638557

[25] Longo PC. Dimensional changes of facial soft tissue associated with rapid palatal expansion. Ann Arbor, MI: Marquette University (2014). p. 82.

[26] Bazargani F Lund H Magnuson A Ludwig B. Skeletal and dentoalveolar effects using tooth-borne and tooth-bone-borne RME appliances: a randomized controlled trial with 1-year follow-up. Eur J Orthod. (2021) 43(3):245–53. 10.1093/ejo/cjaa040 32761047

[27] Lorkiewicz-Muszyńska D Kociemba W Rewekant A Sroka A Jończyk-Potoczna K Patelska-Banaszewska M Development of the maxillary sinus from birth to age 18. Postnatal growth pattern. Int J Pediatr Otorhinolaryngol. (2015) 79(9):1393–400. 10.1016/j.ijporl.2015.05.032 26162781

[28] Pangrazio-Kulbersh V Wine P Haughey M Pajtas B Kaczynski R. Cone beam computed tomography evaluation of changes in the naso-maxillary complex associated with two types of maxillary expanders. Angle Orthod. (2012) 82(3):448–57. 10.2319/072211-464.1 22032536 PMC8865835

[29] Xi T Laskowska M van de Voort N Ghaeminia H Pawlak W Bergé S The effects of surgically assisted rapid maxillary expansion (SARME) on the dental show and chin projection. J Craniomaxillofac Surg. (2017) 45(11):1835–41. 10.1016/j.jcms.2017.08.023 28935486

[30] Zhao T Hua F He H. Rapid maxillary expansion may increase the upper airway volume of growing patients with maxillary transverse deficiency. J Evid Based Dent Pract. (2021) 21(3):101579. 10.1016/j.jebdp.2021.101579 34479662

[31] Lo Giudice A Ronsivalle V Gastaldi G Leonardi R. Assessment of the accuracy of imaging software for 3D rendering of the upper airway, usable in orthodontic and craniofacial clinical settings. Prog Orthod. (2022) 23(1):22. 10.1186/s40510-022-00413-8 35691961 PMC9189077

[32] Giudice AL Ronsivalle V Spampinato C Leonardi R. Fully automatic segmentation of the mandible based on convolutional neural networks (CNNs). Orthod Craniofac Res. (2021) 24(Suppl 2):100–7. 10.1111/ocr.12536 34553817

