# Supplementary material for: Rapid maxillary expansion treatment increases mid-facial depth in early mixed dentition
Source: Front Pediatr. 2023 Feb 1;10:1028968. doi: 10.3389/fped.2022.1028968 (PMC9929059; doi:10.3389/fped.2022.1028968)
Supplement: Supplementary file 1 [file Datasheet1.pdf]

Supplementary Material

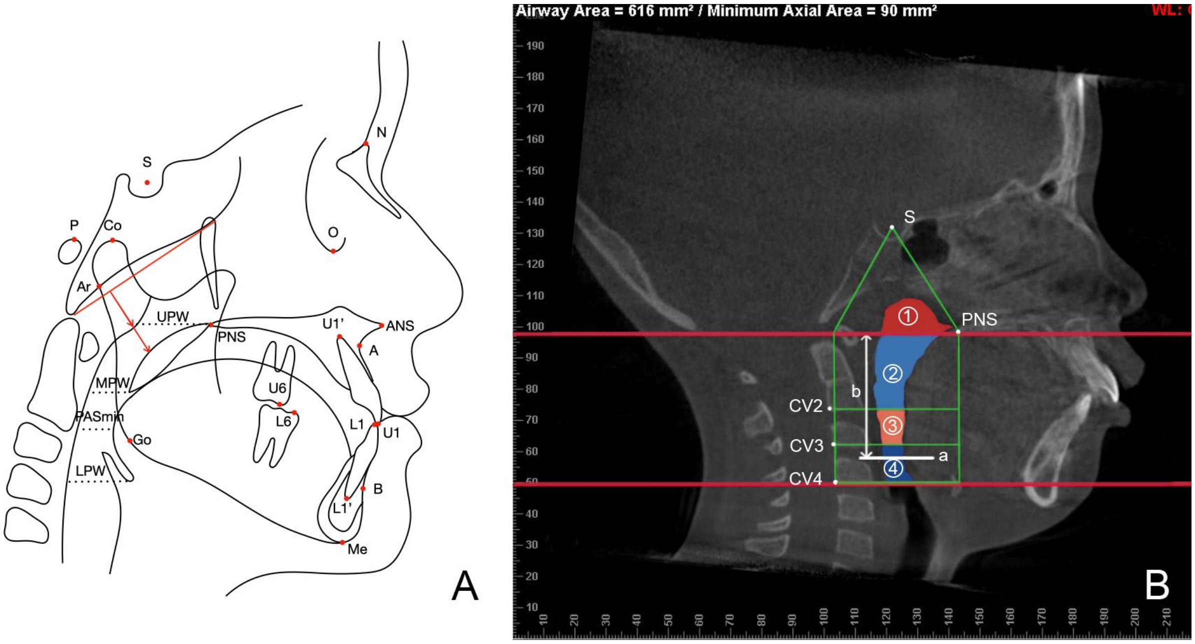

Supplementary Figure 1. Cephalometric measurements of upper airway. (A) PM-UPW, depth of the nasopharyngeal airway space from pterygo-maxillare to upper pharyngeal wall; U-MPW, depth of the retropalatal oropharyngeal airway space from uvula to middle pharyngeal wall; V-LPW, depth of the hypopharyngeal airway space from vallecula to lower pharyngeal wall; PASmin, the shortest distance between the base of the tongue and the posterior pharyngeal wall. (B) Illustration of the upper airway. CV2, CV3, CV4: the lowest point of posterior margin of the 2nd, 3rd and 4th cervical vertebra; ① nasopharynx volume; ② upper oropharyngeal volume; ③ middle oropharyngeal volume;④ lower oropharyngeal volume; a. minimal cross-sectional area; b. the distance from the MCA to PP plane.

Supplementary Table 1． Comparison of the upper airway changes between T0 and T1

Measurements	T0		T1		T1-T0		P-value
	Mean	Std. Dev	Mean	Std. Dev	Mean	Std. Dev	
Airway depth (mm)							
PM-UPW	18.15	4.03	19.61	3.49	1.56	3.03	0.011*
U-MPW	9.59	2.28	10.52	2.93	0.96	2.15	0.026*

V-LPW	10.04	3.15	11.75	3.31	1.49	4.03	0.015*
PASmin	7.02	2.51	7.98	2.88	1.00	3.15	0.11
Airway volume (mm3)							
NV	2704.66	886.36	3164.13	1002.92	458.87	599.42	0.000*
UOV	5763.00	2423.65	7019.65	3154.90	1560.38	2365.64	0.014*
MOV	1977.91	782.35	2325.46	945.05	477.78	851.46	0.047*
LOV	2107.05	612.28	2526.07	746.65	474.85	724.23	0.005*
TOV	11675.69	4385.02	14103.77	5627.56	2932.99	3864.82	0.006*
Minimum cross-sectional							
MCA (mm^2)	112.30	53.85	119.84	84.67	29.91	65.82	0.614
MCA to PP (mm)	23.50	8.05	27.34	9.25	3.63	9,64	0.027*

Std. Dev, standard deviation; T0, before treatment; T1, 6 months after retention; PM-UPW: nasopharyngeal airway depth; U-MPW, oropharyngeal airway depth; V-LPW, hypopharyngeal airway depth; PASmin, minimal pharyngeal airway depth; NV, nasopharynx volume; UOV, upper oropharyngeal volume; MOV, middle oropharyngeal volume; LOV, lower oropharyngeal volume; TOV, total oropharyngeal volume; MCA, minimal cross-sectional area; MCA-PP, the distance from the MCA to PP plane.

* Wilcoxon signed-rank test: $P \leqslant 0.05$.
